# Skull Base Tumors: The Equilibrium between Curation and Preservation

**DOI:** 10.3390/cancers15102829

**Published:** 2023-05-19

**Authors:** Max E. Keizer, Henricus P. M. Kunst, Yasin Temel

**Affiliations:** 1 Department of Neurosurgery, Maastricht University Medical Center, 6229 HX Maastricht, The Netherlands; 2 Academic Alliance for Skull Base Pathology, Maastricht and Radboud University Medical Centers, 6229 HX Maastricht, The Netherlands; dirk.kunst@mumc.nl; 3 Department of Otorhinolaryngology and Head & Neck Surgery, Radboudumc, 6525 GA Nijmegen, The Netherlands; 4 Department of Otorhinolaryngology, Maastricht University Medical Center, 6525 GA Maastricht, The Netherlands

Tumors located at the skull base constitute a particular challenge for medical teams. This is linked to the complex anatomy and the presence of functionally important structures, including the cochlea, vestibulum, cranial nerves, blood vessels, and brainstem. Typical skull base tumors are cholesteatoma, chordoma, chondrosarcoma, meningioma, paraganglioma, schwannoma, and cholesterol granuloma. The surgical treatment of such tumors is often located in the territory of both the ENT surgeon and neurosurgeon. Therefore, in most skull base teams, ENT surgeons and neurosurgeons work and operate together. A multi-disciplinary approach is necessary in order to be able to provide a high level of care and includes the presence of a radiologist, pathologist, and radiotherapist specialized in skull base pathology. Historically, skull base tumor surgery was aggressive, mainly striving for complete resection. However, the modern treatment goal has shifted towards the preservation of neurological function, often resulting in less aggressive tumor removal. With the arrival of new radiotherapy options, such as proton beam therapy, tumor control may be achieved without placing the patient at risk of severe surgical complications. In this Special Issue; six original articles and one systematic review provide an overview of recent developments in the field ranging from experimental to clinical studies.

Meningioma are the most common intracranial tumors [1]. In general they are slow growing, benign pathologies; however, depending on the location and interaction with critical neurovascular structures, their impact on life may be severe [2]. A radical resection is historically a determining factor in achieving curation [3]. However, skull base meningioma is not always eligible for complete resection, leading to a higher risk of recurrence and requiring strict observation [2].

Intraoperative neuronavigation and, more recently, augmented reality (AR) are tools that may aid in achieving the aforementioned surgical goals. Pojskic et al. demonstrate the feasibility of AR in guiding safe skull base meningioma resection [4]. Furthermore, it may play a role in training young neurosurgeons in the field of skull base surgery or allow the surgeon rehearse its operative plan. Future studies must provide more details on the effect of the functional outcome and tumor control in relation to possible additional costs, extended operative time, and general applicability.

The meningioma recurrence rate is strongly related to the level of resection and WHO grading [1,3]. There is a need for additional recurrence predictors to guide follow-up or adjuvant therapy decision making. Lampmann et al. found a similar prognostic rate using the Molecular Immunology Borstel 1 (MIB-1) labeling index in comparison to the already applied mitotic count (MC) [5]. Interestingly, in this cohort MIB-1 demonstrated a high sensitivity of 89% at a threshold of 4.75 (Youden index) and low specificity (46%) for predicting recurrence. In contrast, MC at a threshold of 6.5 is more specific (98%) and less sensitive (22%) for tumor recurrence. Combining both analyses might complement each other in predicting tumor behavior; however, there are no data provided underlining this statement.

Vestibular schwannoma (VS) is the most common benign tumor of the cerebellopontine angle [6]. Its growth behavior is receiving increased attention with numerous studies on possible factors involved in tumor progression [7,8,9,10]. One hypothesis is based on an inflammatory component being predominant in growing VS. Leisz et al. reported interesting results in their cohort, demonstrating a positive correlation between macrophage marker (CD68 and CD163) expression and VS volume and growth rate [11]. However, COX2, Ki-67, and VEGF expression were all negatively correlated with large VS and fast-growing tumors, contradicting previous studies [12,13]. One explanation is an increased recruitment of M2 macrophages in active, growing VS, leading to swelling and escalating growth [14]. Another theory is increased pro-oncogenic tumor-associated macrophages (TAM) that are suspected tumor growth promoters, expressing growth factors, cytokines, and chemokines [15]. The tumor microenvironment of VS needs to be further elucidated in future studies.

Cholesterol granuloma comprises benign cystic lesions that may occur in the temporal bone [16]. It is an extremely rare pathology, which is often an incidental finding during cranial imaging [16]. De Bock et al. describe their multicenter experience on the management of these tumors [17]. The majority of patients could be treated conservatively with a wait and scan policy. Surgical candidates demonstrated symptom progression mostly in combination with granuloma growth. All but one surgically treated patient demonstrated symptom improvement. They conclude that a wait and scan policy is a safe option for stable lesions with acceptable symptoms. Surgery demonstrates successful symptom relief with limited adverse events.

We stay at the lateral skull base and move onto jugular foramen schwannoma (JFS): rare tumors originating from the lower cranial nerves that are often associated with severe neurological symptoms [18]. There is no evidence-based superior treatment, and there are advocates for both surgery and radiotherapy [19,20,21,22]. Aftahy et al. strengthens the discussion by providing their single center experience on surgery for JFS, suggesting to keep it simple and straightforward by relying on classic skull base approaches [23]. Their relatively small cohort demonstrates excellent tumor resection rates with reasonable adverse events using the retrosigmoid and the far lateral, transcondylar approach. Limited follow-up provides no details on long-term results regarding tumor control and functional outcome. They conclude with a specific role for stereotactic radiotherapy for small lesions without significant brainstem compression and primary surgery for tumors with compression.

Yildiz et al. advocates opposing treatment strategies in the management of temporal bone paraganglioma (TBP) [24]. In their very long-term follow-up, they achieved excellent tumor control and functional outcome rates after surgery for small (Fisch A and B) TBP. In contrast, the larger tumors (Fisch C and D) showed more recurrence and new cranial nerve deficits after primary surgery in comparison to primary stereotactic or fractionated radiotherapy.

The final paper in this Special Issue is a systematic review on skull base chondrosarcoma (SBC) [25]. A total of 33 studies involving 1307 patients were analyzed. The main presenting symptoms were diplopia and headache. Cranial nerve neuropathies were reported in 62%, most commonly the fifth and sixth cranial nerves. The vast majority underwent surgical resection (93.3%), of which 89.5% underwent a craniotomy and 9.1% an endoscopic approach. Gross total, subtotal, and partial resection were achieved in 37.8%, 45.7%, and 16.5% of patients, respectively. Adjuvant radiotherapy was administered in 77.9% of all cases: photon (32.2%), proton (50%), and carbon-based radiotherapy (10.2%) more specifically.

Cerebrospinal fluid leaks requiring revision surgery were observed in 6.5% of post-surgical patients. In total, 10.6% suffered from permanent post-operative complications and 15.8% suffered from transient complications; in both groups, mainly new cranial nerve deficits were observed. Regarding radiotherapy complications, a total of 30.7% experienced severe adverse events, predominantly hypopituitarism (15.4%), hearing loss (7.1%), and cerebral necrosis (3.7%).

At follow-up, symptom improvement was recorded in 46.7% after treatment with no differences between radiotherapy types. Over half of the lesions (58.3%) showed a stable tumor volume, 27.1% demonstrated shrinkage, and 14.6% recurred after a median follow-up time of 67 months (range of 0.1–376). Finally, 5-year and 10-year overall survival rates were 94% and 84%, respectively.

In conclusion, these studies portray the ongoing challenges skull base teams face in treating their patients. A paradigm shift started a few decades back with the field turning from debilitating complete surgical resections towards patient-tailored multimodal treatment strategies. We believe that the future management of skull base tumors will become increasingly based on molecular tumor characteristics, guiding treatment choices and not solely relying on anatomical and surgical parameters. 
